# Erie County Medical Society

**Published:** 1875-01

**Authors:** 


### Editorial.

### Annual Meeting of the Erie County Medical Society.

The Annual Meeting of the Erie County Medical Society, was held at the Buffalo Medical College, Tuesday, January 12th, 1875.

The meeting was called to order by the President, Dr. Thomas Lothrop, and Dr. E. N. Brush, elected Secretary *pro tem.* The roll was called by the Secretary, and a large number of members answered to their names. The

Treasurer presented his report, which showed a balance on hand of $18.51. The report was referred to an auditing committee, and being found correct was filed. The President appointed as a committee on credentials, Drs. HOPKINS, O'BRIEN and ABBOTT, who reported in favor of the admission of Drs, Lucien Howe, O. C. Shaw, J. P. Frink, and P. Sonnick, upon compliance with the by-laws of the Society.

Dr. H. R. HOPKINS, chairman of the primary board, presented the report of that body, and gave a list of students who had appeared before them for examination preparatory to entering upon the study of medicine. The report was received and placed on file.

Dr. C. C. F. GAY, chairman of the committee on Hygiene, submitted the report of that committee. The report was an interesting and valuable review of the hygienic condition of the city and county, and embodied replies to the questions prepared by the chairman of the committee on Hygiene of the State Medical Society; its length prevents us from publishing it in full. The report was received, and upon motion of Dr. STORK, a copy was referred to the public press for publication.

The Secretary, Dr. D. E. CHACE, next presented the report of the board of censors, which was received and placed on file.

The following preamble and resolutions were offered, and upon motion of Dr. JAS. P. WHITE, were unanimously adopted:

Whereas, It is believed that a movement has been inaugurated having for its object to abrogate or practically nullify the clause in the present law for the prevention of cruelty to animals, which declares that "Nothing in this act shall be construed to prohibit or interfere with any properly conducted scientific experiments or investigations;" therefore

*Resolved*, That in the opinion of this Society, investigations in physiology and path logy, by means of experiments on animals, are and have been the most fruitful source of increased knowledge with regard to the causes of disease, and its means of prevention and cure;

II. That these experiments and investigations are habitually carried on by medical men without cruelty, nearly always without suffering to the animals employed, and never with any wanton infliction of pain or distress;

III. That authoritative interference, inspection or control of these investigations, by persons unacquainted with their methods and objects, would necessarily defeat their aims and practically prevent their performance.

IV. That the suppression of experimental investigations of this nature, would arrest the progress of physiological science, would obstruct the acquisition of knowledge in regard to the causes of disease, and would be accordingly a disaster to the profession of medicine and to the welfare of mankind; also

*Resolved*, That for these reasons the members of this Society earnestly hope that the leagal provision now existing, which recognizes the importance of such investigations, will be continued in full force and effect.

The Society then took a recess until three P. M.

At three o'clock the Society was again called to order by the President Upon motion the report of the committee on Hygiene was referred to Dr A. N. BELL, chairman of the committee on Hygiene of the State Medical Society, and the secretary directed to forward a copy. Dr. BRUSH moved that the same committee be continued for another year. The credentials of Dr. Isaac G. Wheeler, were referred to a committee consisting of Drs. Brush Gould and Harding, who recommended his admission to membership upon compliance with the by-laws. Dr. E. T. Dorland read the annual essay, which was referred for publication to the Buffalo Medical Journal.

After the transaction of some miscellaneous business, the Society proceeded to the election of officers for the ensuing year, the result was as follows:

*President*—Dr. J. C. Cronyn, of Buffalo.
*Vice President*—Dr. R. S. Myers, of Clarence.
*Secretary*—Dr. D. E. Chace, of Buffalo.
*Treasurer*—Dr. W. C. Phelps, "

*Censors.*—Dr. E. Stork, Dr. D. E. Chace, Dr. A. H. Briggs, Dr. E. R. Barnes, Dr. C. C. Wyckoff.

*Primary Board.*—Dr. H. R. Hopkins, Dr. M. B. Folwell, Dr. M. Willoughby. Committee on Hygiene holding over Dr. C. C. F. Gay, Dr. W. Gould, Dr. R. S. Myers and Dr. T. M. Johnson.

Dr. C. C. F. Gay was appointed essayist for the next meeting, with Dr. Lucien Howe, alternate.

The retiring President, Dr. Lothrop, made a brief address in which he recommended a change in the order of business, his address was somewhat shorter than we should have wished, but was listened to with marked attention, at its conclusion a vote of thanks was tendered to the President and retiring officers.

Dr. H. R. Hopkins gave notice of intention to amend the by-laws so as to make the Presidents address third in the order of business for the annual meeting; some unfinished business was transacted and the Society adjourned.

